# Performative paternalism

**DOI:** 10.1007/s13194-025-00651-7

**Published:** 2025-04-14

**Authors:** Jakob Ortmann

**Affiliations:** Centre for Ethics and Law in the Life Sciences (CELLS), Philosophische Fakultät, Leibniz University of Hannover, Otto-Brenner-Strasse 1, 30159 Hannover, Germany

**Keywords:** Performativity, Reflexivity, Values in science, Politics, Paternalism

## Abstract

Performativity of science refers to the phenomenon that the dissemination of scientific conceptualisations can sometimes affect their target systems or referents. A widely held view in the literature is that scientists ought not to deliberately deploy performative models or theories with the aim of eliciting desirable changes in their target systems. This paper has three aims. First, I cast and defend this received view as a worry about autonomy-infringing paternalism and, to that end, develop a taxonomy of the harms it can impose. Second, I consider various approaches to this worry within the extant literature and argue that these offer only unsatisfactory responses. Third, I propose two positive claims. Manipulation of target systems is (a) not inherently paternalist and can be unproblematic, and is (b) sometimes paternalist, but whenever such paternalism is inescapable, it has got to be justifiable. I generalise an example of modelling international climate change coordination to develop this point.

## Introduction

It is a well-appreciated fact in the sociology and philosophy of science that scientific conceptualisations, broadly construed, do not merely *depict* their objects of study, but can sometimes *change* them.^1^ For example, if a prominent economist publicly announces that they think a bank run is likely, this announcement may very well magnify the probability of the bank run actually occurring, making it a “self-fulfilling prophecy” (Merton, 1948). More recently, related effects have been dubbed as *performativity* of science (Callon, 1998; MacKenzie & Millo, 2003), but various terminologies co-exist.^2^ Despite much heterogeneity, a common thread among the broader literature is that science’s causal influence on the very targets it is studying tends to create *epistemic* and *ethical* issues (see e.g. Marchionni et al., 2024). Epistemic issues, because it is not clear whether in such cases there even exists “a stable object […] to have knowledge about” (Hacking, 1995, 61). In the economist’s case, for instance, one might be wondering whether there would have been a bank run was it not for the prediction itself. And if not, could the prediction have been ‘true’ in any meaningful sense? Ethical issues, because it is not self-evident what the scientist should do with such unsought transformative power; for example, should the economist have kept quiet about a possible bank run or did they have a responsibility to warn the public?

Across disciplines, various strategies have been proposed to handle both problems, the ethical and the epistemic (see e.g. Khosrowi, 2023). According to what I will call here the *strategy of manipulation*, we need not be too troubled by epistemic inadequacies caused by performativity. Instead, the argument goes, we could focus on the consequences of performativity from an ethical point of view and use performativity as a tool for eliciting changes or states within the target that are deemed desirable. To continue the simplified example, proponents of this strategy might suggest that the economist ought to hold back their prediction, even if they thought it was true, with the specific purpose of eliciting the desired outcome of averting a bank run. Variants of this strategy have been applied to epidemiology (van Basshuysen et al., 2021), climate change negotiations (Ortmann & Veit, 2023), decolonial activist research (Koskinen, 2022), human kinds more generally (Godman & Marchionni, 2022) and more.

Perhaps unsurprisingly, the strategy of manipulation has provoked various criticisms questioning its overall legitimacy. Specifically, it gave rise to a concern I will dub *performative paternalism*. Winsberg and Harvard (2022, 516), for instance, argued that using performativity as a tool to drive certain outcomes would be unjust and deceitful. Concealed as a value-neutral stance of just “following the science”, deliberate deployment of performative models would impose scientists’ own value judgments on society, effectively depriving individuals and policymakers of the ability to make their own informed value decisions. By adopting a manipulative role, scientists act outside their legitimate competencies and duties, which involve providing society with predictions, explanations, etc., that are, to the largest extent possible, apolitical and uninfluenced by the scientists’ own values. As such, they argue, performativity “is never a legitimate purpose for a model “, and “a serious threat to democratic decision making “. Khosrowi (2023) and van Basshuysen et al., (2021, 122–123) share a similar worry. They agree that “[m]odels are widely considered to be epistemic instruments” (Khosrowi, 2023, 382), and, as such, should not be misused for the non-epistemic purpose of deceitfully manipulating target systems.

Contra these worries, in this essay, I contend that the ethics of the manipulation strategy are more convoluted. As such, there are conceivable conditions under which the strategy of manipulation can be justified. Specifically, I argue for two things. (i) Autonomy-infringing deceit is not an inherent feature of the strategy of manipulation and, therefore, it does not have to be paternalistic. (ii) Whenever a paternalistic performative choice is inescapable, making some choice has got to be justifiable. As such, this argument contributes to a growing literature on performativity, which has made notable advances regarding its epistemic aspects but has left ethical and political aspects largely underdeveloped (Marchionni et al., 2024, 8). I proceed as follows.

Section 2 delineates how I intend to use the term performativity for the purposes of this paper. Against the background of a diverse literature surrounding the term, this is a necessary step for internal consistency. Section 3 elaborates the *strategy of manipulation* in more detail. Section 4 develops various critiques that have been raised against the strategy of manipulation and collates them as a worry of *performative paternalism*. Section 5 considers four strategies from the literature aimed at avoiding said harms. Unfortunately, I argue, none of these strategies is individually satisfactory for keeping illegitimate value influences at bay across all cases of performativity. Apart from suffering various practical and conceptual problems, they tend to perpetuate the very same harms their proponents sought to solve. Thus, in Section 6, I revisit the strategy of manipulation and develop a case in support of performative paternalism. The positive claim I propose rests on the acceptance that performative paternalism is, at times, inescapable. Section 7 concludes.

## What is performativity?

Although the meaning of the term performativity, as used above, might appear reasonably straightforward, definitions have notoriously been vague. Judith Butler (1990, xiv), for instance, conceded that it is “difficult to say precisely what performativity is “, and Francesco Guala (2016, 30) acknowledged that performativity is “not […] well-defined”. In this section, I will briefly discuss selected uses of the term in the context of philosophy of science before settling on a working definition for the purposes of this paper.

Initially, the term performativity had nothing to do with science in particular and was introduced by John Austin (1962) in philosophy of language. On Austin’s account, a performative is a verbal utterance that performs an action. To say, “I bet you sixpence it will rain tomorrow” (Austin, 1962, 5) is not a matter of describing something that already exists out there in the world. Rather, to utter the sentence *is* to perform an action of betting. Importantly, unlike constative propositions such as “the door is open”, a performative utterance like “I bet you X” is seldom thought to have a truth value; precisely because it does not describe anything, but it *constitutes* an act – in this case the act of betting. Yet, Austin went on to argue that *all* utterances are performative in this sense, including constative statements: although a constative statement like “the door is open” describes and does not constitute the state of a door, it does perform some other act of, say, asserting a claim or informing someone of something, etc.

While Austin’s contributions kicked off speech-act theory as a research program in its own right (Green, 2021), his neologism “performativity” had been adopted by several other research programs. In the late 1990s, sociologists and philosophers of economics started to adopt the term to examine the influences that economics as a discipline has on the markets it described (Callon, 1998, 2008; MacKenzie & Millo, 2003; Guala, 2005, 2016; Latour, 2005; MacKenzie, 2006; MacKenzie et al., 2007). Although Austin himself never theorised about science as such, according to Michel Callon (2008, 318), there would be no particular reason “to exclude science from [Austin’s] general rule” that *all* utterances were performative. Following their call, the term performativity has now become relatively common (particularly in economics) to refer to the broad notion that science shapes the world in various ways.

But in what ways, specifically? Saying that science changes the world is almost trivially true. Science as a human enterprise does not happen in a vacuum and, for one, has undergone many changes itself, but is also deeply embedded into many changing social practices. To give just one example, gaining knowledge about nuclear fission was certainly a pre-condition for the construction of nuclear bombs, which would be one way to say that theories of nuclear fission contributed to “change” the world – without these theories, the world today would have looked different.

To make this point, however, Callon and colleagues would not have needed to fall back on the neologism of performativity. And indeed, a major focal point of their debates runs deeper. It is the claim that some theories or models (the differences do not matter here) appear to causally interfere specifically with the very phenomenon they are studying. A seminal case study of this effect has been put forward by MacKenzie and Millo (2003), who argued that the adoption of Option Price Theory (Black & Scholes, 1973; Merton, 1973) by traders at the Chicago Board Options Exchange changed market interactions in such a way that they more closely resembled the theory’s prediction than before the theory’s widespread use. As a result, and akin to Austin’s original performatives, saying that the Option Price Theory’s predictions were *true* seems to do injustice to conventional notions of truth, if what it actually did was moulding the world in a particular way such that the world fits its predictions (rather than the other way around, i.e. moulding predictions to fit the world).

Clearly, the downstream effects of the adoption of Option Price Theory elucidate a more specific kind of change compared to the changes brought about by the adoption of theories of nuclear fission. While a theory of nuclear fission does not change the process it refers to, the same has arguably not been true for Option Price Theory. In the following, I will distinguish between the two with the following working definition.^3^ A model, theory or prediction is.

(i) *generically performative* if and only if its dissemination has any impact on the world at all, and it is
(ii) *reflexively performative* if and only if its dissemination has an impact not only on the world, but specifically on the target it is designed to depict such that the changes caused by this impact bear on model-target-fit or predictive capacity.

Understood as such, many (if not all) models or theories are at least generically performative. It is also in this utmost generic sense of performativity that one might be inclined to agree with Callon’s otherwise contentious claim that “all science is performative” (Callon, 2008, 318), which is evidently not true if he means to suggest that all of science would be *reflexively* performative, i.e. that all of science affects the phenomena it studies in ways that bear on model-target-fit or predictive accuracy. Again, recall the downstream effects of models of phenomena like nuclear fission.

There is more to be said, and overall, the ontological and conceptual status of performativity remains contested (Callon & Roth, 2021; Guala, 2005, 2007, 2016; Mäki, 2013; Peled, 2020). For simplicity, in the remainder of this paper, when I talk about performativity, I will exclusively refer to what I have just called reflexive performativity. I will ignore both the generic and Austin’s constitutional forms of performativity while making no claim about whether they are genuine, distinct, analogous, congruent or otherwise. An example of a reflexively performative model is the Option Price Theory, and an example of a reflexively performative prediction is the bank run case. Conceivably involving a range of different causal mechanisms, this is a very broad category. It may or may not share important features with Austin’s performative utterances, but nothing much, I believe, rides on this for the question of whether (reflexive) performative paternalism is justifiable.

## The strategy of manipulation

Previously, I have claimed that (reflexive) performativity gives rise to unique epistemic and ethical problems.^4^ In this section, I will elaborate on these problems and consider a first potential coping strategy to handle them: the *strategy of manipulation*, as I shall call it.

In short, proponents of this strategy hold that sometimes we can deliberately use performative effects to elicit certain outcomes within the referents of our theories or models. This often comes with the aspiration that such a practice helps address the ethical and epistemic challenges posed by performativity. Note that the name of manipulation here is not intended to discredit its proponents from the start. Instead, it capitalises on a useful double meaning: “manipulation” is predominantly used to refer to deceitful forms of social influence. At other times, however, it indicates non-deceitful acts of causal influence; for example when it is said that “an engineer manipulates their machine”. As will be argued later, importantly, both meanings are at issue when evaluating the usage of performativity as a tool to drive social change.

To start, however, let me detail what exactly the epistemic problem is supposed to be.

### The epistemic problem

Consider the following example. During the COVID-19 pandemic, epidemiological models played a key role in informing policymakers and the general public about the current and future state of the ongoing pandemic (van Basshuysen et al., 2021). Naturally, the usefulness of these models has widely been considered to be attached to their *predictive performance* (e.g. Friedman et al., 2021).^5^ Quite reasonably, a model that consistently provides highly inaccurate predictions, e.g. because it significantly overestimates the required capacity of critical care beds, plausibly falls short of meeting its informational objective. For example, it is important for policymakers to gauge how many critical care beds are likely to be required in a given scenario in order to decide between various abatement measures.

However, as van Basshuysen et al. (2021) argue, the very same mitigative measures that are supposed to be sparked by such predictions also have the capacity to alter the pandemic landscape in a way that is not accounted for by said models (see also Friedman et al., 2021). For example, the general public might respond to deteriorating conditions with increased compliance to social distancing policies in ways that have not been anticipated by the modellers.

Clearly, if unaccounted for, such effects can be detrimental to a model’s predictive performance. For example, whereas an unabated pandemic would expectedly show an exponential growth of COVID-19 cases, which is what many models had predicted, the actual incidence has often only grown linearly. Friedman et al., (2021, 9) largely attribute this overestimation to behavioural changes in response to publicised predictions. As such, van Basshuysen et al. (2021) consider this to be a case of (reflexive) performativity: through various causal pathways, the propagation of the models’ predictions causally interfered with the modelling target itself such that model-target-fit was degraded, and predictive performance worsened.

Evidently, such reactions to model predictions prompt an epistemic problem. How are epidemiologists supposed to explain or predict future conditions or draft counter-measures if their model target changes the very moment their works are being circulated? Furthermore, by arguing that the prediction would have been correct had the public not changed their behaviour in response to predictions, any bad model could theoretically be immunised against any later evidence of the actual death toll. Without the counterfactual, such a defence of a potentially bad model can be difficult to evaluate. This creates political downstream issues, too. As van Basshuysen et al. (2021) stress, perceived poor predictive accuracy can undermine the overall credibility of epidemiological models and scientific institutions more broadly. For example, a model that seems to be consistently overestimating infection cases might soon come to be regarded as overly pessimistic, and the scientists promoting it as not trustworthy after all. According to Friedman et al., (2021, 9), continued overestimation that was due to model responses had been a problem for many COVID-19 models.

There is a notable difference between this pandemic case and previous examples. Whereas the COVID-19 case signified a drop in predictive performance, which one might call a *self-defeating prophecy* (van Basshuysen et al., 2021), the bank run and Option Price Theory were more akin to a *self-fulfilling prophecy* given that their circulation led to “improvements” of predictive accuracy. Nevertheless, we can consider the epistemic problems they prompt to be largely analogous: Performativity, as it seems, pushes modelling targets beyond our epistemic access, given that they change through our epistemic efforts to understand them in the first place. Whether this improves or deteriorates predictive accuracy, we cannot seem to get the whole picture of what is truly going on.

### Using performativity as a tool

This prompts an ethical question: what ought the epidemiologist do in such epistemically confounded circumstances? Over the course of this paper, I will consider multiple answers, but according to van Basshuysen et al. (2021), in the case of the COVID-19 models, the answer is: not too much differently! Specifically, they argue that the models’ poor predictive performance need not necessarily be regarded as a problem, but if this means that the models were performative in a desirable way, this could sometimes be regarded as a virtue, too. By contributing to bringing down infection rates, they argue, the effects of performativity, in this particular case, were desirable given that they aligned with the public’s preferences of keeping the pandemic in check. When evaluating a model’s performance in an all-things-considered manner, they argue, we ought to appraise its performative effects, too, and even go so far as regarding performativity as a potential modelling purpose in its own right.

They provide us with an analogy to illustrate their point, and to which I will keep returning. Picture a medical doctor warning a patient about the potentially fatal consequences of their unhealthy lifestyle. The doctor might be unable to predict how the patient will change their behaviour in response to this warning and whether the patient is, in fact, going to die soon, but they are justified in advancing their conditional prediction (“if you live healthier, you will live longer”) with the specific purpose of being performative. In their words:

*“Just as we should not think that a doctor is unqualified because she cannot tell whether a patient would indeed have died of a heart attack counterfactually, or because she cannot accurately forecast how much longer, exactly, a patient would live under a changed exercise regime, we suggest that it is unhelpful to assess the utility of epidemiological models based on their predictive abilities alone. Epidemiological models have been a crucial resource for informing and justifying policy interventions, and may have contributed to shaping both the public’s understanding of the pandemic and their behavioural response to it. The ability to make such performative contributions can be understood as a desirable feature […]” (*van Basshuysen et al., 2021*, 121)*

Casting a model’s performativity as a virtue rather than a vice might be one way of coping with the apparent problems posed by performativity. Instead of being too hung up with its apparently lacking epistemic qualities, one may just deploy the model anyway in an attempt to elicit desirable (social) change. For this reason, I will label van Basshuysen’s and colleagues’ proposal as part of a class of coping strategies that I will refer to as *strategies of manipulation*. I will defend this approach in more detail later, but note for now that there are multiple recent contributions that take on a similar line of argument (Godman & Marchionni, 2022; Koskinen, 2022; Ortmann & Veit, 2023).

As the name of manipulation might suggest, however, the idea of deliberately using models for the non-epistemic aim of nudging target systems towards desired outcomes sparks serious questions of legitimacy. According to a response by Winsberg and Harvard (2022, 514–515), for instance, performativity as a model purpose is deceitful and unjust. Concealed as a value-neutral stance of just “following the science”, deployment of performative models would impose scientists’ own value judgments on society, depriving individuals and policymakers of the ability to make their own informed value decisions. According to them, producing worst-case scenarios that are unlikely to occur just to spur people into certain behaviours is unacceptable. This is because public health interventions (e.g. social distancing), they argue, come with costs, too, and any political deliberation needs to weigh off the costs and benefits of such measures. For such determination processes, the scientist’s role should be restricted to providing society with explanations, predictions and counterfactuals that help to estimate costs and benefits instead of pushing for mitigative measures that they themselves think are right. As such, Winsberg and Harvard (2022, 515) argue, performativity “is never a legitimate purpose for a model “, and “a serious threat to democratic decision making “.

Anticipating and sharing a similar worry, however, van Basshuysen et al. (2021) advance a more nuanced position. Donal Khosrowi (2023, 382), one of the co-authors in van Basshuysen et al. (2021), agrees with Winsberg and Harvard that “[m]odels are widely considered to be epistemic instruments”, which, as such, should not be misused for the non-epistemic purpose of manipulating target systems. Not only would this “raise significant concerns about illegitimate value-influences “, but it also could “severely undermine the epistemic credentials of models” (van Basshuysen et al., 2021, 122). Yet, contra Winsberg and Harvard, van Basshuysen et al. (2021) distinguish between what I will call *ex-ante* and *ex-post* appraisal of a model’s performativity. While they agree that scientists ought to refrain from deliberate deployment of performative models to steer peoples’ future behaviour, they argue we may appraise a model’s performativity *after the fact,* irrespective of whether it has failed at other epistemic tasks (such as prediction). In their words:

*[P]erformativity should only play an evaluative, but not a prescriptive, role in model appraisal. It may figure in judgments concerning whether the downstream performative aspects of models have been desirable or undesirable, but such judgments should not bear on decisions made at the stage of model construction, selection, or deployment—there should be no* wishful modelling*. (*van Basshuysen et al., 2021*, 123; emphasis in orig.)*

The terms *evaluative* and *prescriptive* here need to be understood as implying two strictly distinct temporal orders of things. Khosrowi (2023, 382; emphasis in orig.) makes this more explicit and informs us that their appraisal view “is *backward-looking*: given a model that has been used in such-and-such ways, we consider what differences it made […] and this guides our assessment of its overall goodness”. In turn, the view they dismiss is *forward-looking*. Accordingly, all ex-ante anticipations of performative effects and model choice based on such anticipations should be refrained from, given that this places scientists in an untenably manipulative position.

On a side note, this dismissal of *ex-ante* appraisal seems to introduce an interesting tension within the view of van Basshuysen et al. (2021). As I read their own example, the performative effect of the doctor’s prediction is that, informed by this conditional, the patient is empowered to choose to act in a way that might bear on predictive accuracy, which is overall desirable. However, this clearly appears to be a form of *ex-ante* appraisal of performativity, which, by virtue of being desirable, also seems prescriptive in the sense that this is what doctors *should* do. Such performativity of the doctor’s conditional prediction undeniably plays a crucial part in how the doctor chooses to construct, select and deploy their prediction.

I will scrutinise this *ex-post-only* restriction later in more detail. However, first, I will turn my attention to the concern that the manipulation strategy introduces illegitimate value influences. Although there are diverging positions on whether at least *ex-post* appraisal of performativity is justifiable, both sides are (or at least seek to be) clear about the wrongfulness of using performativity *ex-ante* to manipulate target systems. Winsberg and Harvard discard it out of hand, while Basshuysen et al. are clearly worried, too. In the following section, I will attempt to strengthen these arguments and spell out in more detail what precisely one might consider wrongful about it. In particular, I will point out four distinct types of wrongs that render the strategy objectionable, contributing to an adverse form of *performative paternalism*.

Subsequently, in Section 5, I examine four strategies for avoiding performative paternalism, yet find them lacking in their ability to address the identified wrongs: among other problems, they tend to perpetuate the same harms they seek to solve. Thus, Section 6 revisits the strategy of manipulation and develops a positive view in defence of the strategy of manipulation and paternalist choice-making among performative models.

## Performative paternalism and its wrongs

If the strategy of manipulation is illegitimate, what exactly makes it so? Reading a bit between the lines, I take it that, collectively, the authors above touch upon at least four aspects that might be considered wrongful about (ex-ante) using performativity to drive social change. I will call these aspects (1) *act obfuscation*, (2) *act appropriation*, (3) *target obfuscation*, and (4) *target outcome*. Together, I take it, these aspects yield the strategy of manipulation to be a harmful instance of what I will call *performative paternalism.*

First, *act obfuscation*. By advancing a performative model while, at the same time, not disclosing this as an attempt to steer a model’s target system in certain directions, scientists appear to unjustly cover up the true act they are performing. At face value, they pretend to offer a standard descriptive, explanatory, or predictive service to society. In reality, however, they act to spur people into certain actions. Act obfuscation of this kind, one might reasonably argue, is harmful and something scientists ought not to engage in.

Second, *act appropriation*. One might think that certain actors like politicians, central bankers, entrepreneurs (or other agents who quite obviously work towards changing and overtly manipulating things in the world) are licensed to deliberately try to deploy performative predictions. For example, a politician’s call-out that “we will win the war” is a very obvious propaganda attempt to induce a self-fulfilling prophecy. In a similar vein, Lepoutre (2024) recently argued that political figures are sometimes justified to deliberately promote falsehoods when trying to mobilise people, for example when it is obvious to the public that their claims are false. Similarly, publicly facing agents like central banks might be licensed, if not mandated, to deliberately assert predictions with the specific purpose of anchoring inflation expectations (Khosrowi, 2023).^6^ Scientists, one might argue, have no license to do this, and by deliberately deploying performative models, they overstep their competencies. As such, the scientist’s role, as the above authors seem to imply, ought to be largely apolitical. Although van Basshuysen et al. (2021) and Winsberg and Harvard (2022, 515) concede that some value influences in scientific practices are inescapable, they both take for granted a shared view that scientists should provide society with good-faith estimates of what *is* the case, rather than work towards what scientists themselves think *ought* to be the case. By violating this restriction when (ex-ante) using performativity as a tool, scientists unjustly assume responsibilities typically reserved for other decision-makers such as policymakers.

Third, *target obfuscation*. Epidemiological models assume various aspects of a pathogen and its environment. As van Basshuysen et al. (2021) argue, they did not model all relevant policy or individual responses. As such, performative models seem to misrepresent their target in a way that might be considered wrongful if these models are deliberately deployed to steer their targets. For example, if one were to disagree with the epidemiologists about the acceptable costs of mitigation efforts, one might consider too-pessimistic predictions to be close to outright lies – the model is false and incomplete precisely because it does not account for all aspects that really matter to gauge future pandemic development, but it is propagated nevertheless. Winsberg and Harvard seem to argue in this direction when they claim that.

*[t]o build an epidemiological model for the purpose of performativity, for example by deliberately producing ‘worst-case scenarios’, is to stack the deck in favour of certain results of a cost-benefit analysis, rather than to perform one. (*Winsberg & Harvard, 2022*, 515)*

Lastly, there is what I call a worry on *target outcome,* capturing the concern that stakeholders might have conflicting views on what constitutes beneficial results of performative model deployment. If there are conflicting views on this and the performative model is deployed nevertheless, without adequate political deliberation, then at least one party’s preferences could thereby get unjustly overruled. This potentially makes performative model choice highly political. Although van Basshuysen et al. (2021) argue that the performative effects of the COVID-19 models had largely been in alignment with public interests given that they induced mitigative measures, Winsberg and Harvard (2022, 515) object and argue that “reasonable people could disagree over whether the costs [of mitigative measures] were worth the benefits that they provided “. Among others, this is a disagreement on what should have been the consequence or outcome of a performative model in the first place (i.e. should mitigative measures have been more drastic or less?), which can lead to scientists actively contributing to harmful conditions for certain individuals and overruling their will.

Taken together, all these four harmful aspects contribute to what one might dub *performative paternalism*. Let me take both parts of this label apart. I take paternalism to mean the following:

*A person P1 acts paternalistically if they non-consensually make decisions on behalf of one or more persons P2 to allegedly increase P2’s well-being, whereas P2 may or may not agree with whether P1’s decisions do, in fact, increase P2’s wellbeing.*^7^

In its performative variety, then, such paternalism manifests in the manipulation strategy as follows, with the four harms reinforcing the overall wrongfulness of paternalism in a manner more particular to the problem of performativity:

First, by virtue of being geared towards social change that is deemed desirable as judged by the scientists, we can consider ex-ante deployment to be an act of deciding on behalf of the well-being of other people irrespective of their agreement.^8^ In the COVID case, if models had been used ex-ante as a tool to drive change, P1 would likely have been epidemiologists with sufficient outreach (or other prominent figures who potentially could have made a serious attempt to deploy performative predictions), and they would have done so on behalf of the supposed well-being of the general population (P2). Second, if act obfuscation or act appropriation hold, then the no-consent condition is arguably fulfilled, too: hiding an act which one might not be licensed to perform is one way of performing an act non-consensually. Third, regarding target outcome, the population P2 may or may not agree with whether their well-being has increased. Fourth, target obfuscation also carries a non-consensual element, especially if it is indeed akin to an outright lie (as Winsberg and Harvard suggest). As a result, by virtue of its obfuscatory character, performative paternalism appears to cut into people’s autonomy, given that their decision-making basis of what to expect and whom to trust is skewed.

Taken together, by being paternalistic and harmful four-fold, the manipulation strategy does not initially seem particularly appealing.

## Four unsatisfactory responses

Against this background, in this section, I will consider four attempts from the literature to cope with the worry of performative paternalism. I will find all of them to be unsatisfactory in differing regards, given that they tend to reproduce the very same harms they are supposed to eradicate. Thus, in Section 6, I revisit the strategy of manipulation and develop a case in support of performative paternalism.

### Ex-post-only appraisal

The first response is offered by van Basshuysen et al. (2021) themselves. Recall their claim that although *ex-ante* deployment of a performative model would be illegitimate, *ex-post* appraisal of performative effects could be justified nevertheless. Accordingly, although COVID-19 models had poor predictive accuracy, we could retrospectively regard them as successful due to their welcomed performative effects. As such, they argued, as long as we restrict appraisal to the retrospective ex-post kind, performativity could be regarded as a legitimate modelling purpose in its own right, too.

However, what exactly, one might ask, is a retrospective modelling purpose supposed to be? In later publications, van Basshuysen (2023) and Khosrowi (2023) draw their notion of a model purpose from the *adequacy-for-purpos*e view of Wendy Parker (2020). According to Parker, different models can be employed for different purposes, and model evaluation involves determining whether the model in question is adequate to achieve the given purpose. Importantly, in Parker’s view, a model purpose need not only be restricted to epistemic purposes (e.g. prediction or explanation) but can also serve practical purposes (e.g. making profits or saving a population from a natural hazard). Although Parker contends that such practical purposes usually break down to epistemic purposes, Winsberg and Harvard (2022, 515) are right in pointing out that models, in principle, could “be used for almost any purpose—displayed as a work of art, incorporated into one’s spiritual practice” and so on. According to van Basshuysen (2023), performativity is one such non-epistemic practical purpose.

The primary goal of Basshuysen et al.’s move to exclude *ex-ante* anticipation of performative effects has been to avoid issues of illegitimate (paternalist) value influences. Admittedly, given that looking back into the past will not change the past, one might think that this move is successful in doing so. However, I remain sceptical of whether this approach can rectify the strategy of manipulation. Apart from the fact that there seem to be tensions regarding their own choice of examples (as remarked earlier), it remains unclear how Basshuysen et al. imagine this to work in practice. As I understand the term, any talk of “purposes” seems to only ever make sense in an *ex-ante* context. A “purpose is a goal” (Parker, 2020, 460) and goals usually lie in the future. One cannot have a goal today to go to bed early yesterday; analogously, one cannot have a goal today that one’s model has been performative last year. Either it has been performative, or it has not, but then this is not a matter of goals but a matter of fact.

On a more charitable reading, the term “purpose” might simply be misplaced. What they appear to have in mind is a two-step process. First, develop a model that adheres to whatever epistemic purposes are deemed relevant at the moment, and second, after everything is over, evaluate whether and to what degree of desirability the model had been performative. Such *ex-post* analysis, I agree, is certainly an interesting task for a whole array of conceivable non-manipulative purposes.

However, deliberate self-restriction to *ex-post-only* evaluation (of what then hopefully will have been desirable performative effects) runs the risk of an equally bad, if not worse, form of target obfuscation: *ex-ante ignorance*. If a scientist has reason to believe that their work is likely to be performative, as might be the case for the medical doctor as well as for the epidemiological models, it is a strong if not impossible requirement to demand that scientists should simply *pretend* not to expect performative effects or cease to make educated guesses about whether these effects are aligned with the public’s values. Not only could this have devastating effects in case the performative effects do not turn out to be desirable after all, in effect artificially confining scientists to the mere hope that performative effects will be acceptable, but it also yields similar issues of illegitimate value influences stemming from deliberately misrepresenting a target: if it is true that something is a self-fulfilling prophecy, for example, it is deceitful, too, to frame it as an unmalleable necessity (I will develop an example of this beginning in Section 6).

I do not have the space to develop these arguments further here, but I will assume, for now, that both the ex-ante and the ex-post version of the strategy of manipulation appear contentious. The former appears paternalistic, the latter unworkable. This begs the question: Are there any other proposals for dealing with performativity in scientific practice? Enter the strategy of endogenisation.

### Endogenisation

Until now, I have largely taken for granted that performativity gives rise to unique epistemic and ethical problems. The sceptical reader might not have agreed. What, one might ask, should be so special about it as a social phenomenon that one could not gain knowledge about its mechanisms and predict its outcomes? This is precisely the intuition of the second coping strategy I will consider. Borrowing from van Basshuysen et al. (2021) and Khosrowi (2023)*,* I will refer to it as the *strategy of endogenisation* of performative effects.

In economics, endogenisation usually refers to the act of including a variable into a given model whose value is determined by relationships specified within the model (see e.g. Wooldridge, 2016). Analogously, in cases of reflexive performativity, proponents of the strategy of endogenisation suggest including performative effects as an explanatory factor in their model itself.^9^

Consider a simplified epidemiological model that predicts the incidence $I$ as a function of time $t$. This involves some function $F$, which models various aspects of the pathogen and its environment, and so on, as well as some error term $e$.

1 $$I\left(t\right)=F\left(t\right)+e$$

Now assume the predictions of model (1) would be circulated and people would start to alter their behaviour significantly. These effects are not captured in $F(t)$, with the result that any prediction $I\left(t\right)$ is now biased. Endogenising performative effects here would mean introducing an additional term that explicitly models these effects. To do that, one might assume an additional function *P,* which takes as a parameter the predicted incidence *I* of the time period $t+1$*.* Function* P* explicitly models the influence that behavioural responses have on model predictions.

2 $$I\left(t\right)=F\left(t\right)+P\left( I\left(t+1\right) \right)+e$$

In model (1*), we have now depicted a recursive feedback loop according to which model predictions $I(t+1)$ feed back into the actual incidence $I(t)$. If done right, one might hope, we thereby have increased model-world-fit and improved predictive accuracy. For short-term predictions, one might then go on to compute $I(t)$ for the desired range of $t$ and for long-term predictions, one might try to solve for an equilibrium of $I$. According to Friedman et al., (2021, 9), schematically, such explicit modelling of model response feedback is precisely what recent epidemiological models have sought to implement more rigorously.^10^

Endogenisation seems attractive for multiple reasons. First, it promises an improved understanding of performativity itself and the multiplicity of causal pathways by which it manifests – for example, be it via policymaking and shared expectations (van Basshuysen et al., 2021), social status (Laimann, 2020), or other candidates proposed in the literature, such as discourses (Callon, 2008), constructed cultural environments (Mallon, 2016), cultural reproduction (Godman, 2020), and so on. To grapple with a phenomenon, we must gear our epistemic tools towards it, and building and testing models that explain performative effects seems, at the very least, like a good-faith effort to achieve exactly that. Thus, ideally, we end up with conceptualisations of target systems which are not challenged by changes due to performativity, but for which we can explain (and potentially even predict) such changes.

Secondly, it might appear as if endogenisation allows scientists to circumvent performativity altogether, including the paternalist problems it brings. When endogenising, it seems, scientists need not choose whether or not they agree with to-be-expected performative effects; instead, they construct models that incorporate, explain and predict these effects, deferring political deliberation on what outcome should be pursued to other actors.

As such, one might think that endogenisation kills two birds with one stone. (a) It counters performativity’s apparent epistemic threats; targets subject to performativity do not stay beyond our epistemic access, instead we can model and understand their changes. (b) Endogenisation appears to provide a simple answer to the ethical question of what scientists ought to do in the face of performativity if they are to avoid illegitimate value influences: they ought to endogenise and stop promoting performative models in a manipulative manner. Despite such reassuring sentiments, however, the strategy of endogenisation suffers a set of problems, too, that render it insufficient to handle the worries of performative paternalism in practice and in principle. I will briefly highlight four arguments.

First, it may sometimes be impossible in practice to pull off endogenisation (van Basshuysen et al., 2021, 120). One might imagine a lack of relevant data, an increased need for resources, highly fragile causal relationships that are difficult to track, and so on. If endogenisation indeed were the dominant strategy of coping with reflexive performativity, then one would require at least some guidance on handling situations wherever we are unable to execute it – guidance currently lacking in the extant literature.

Second, in the literature on values in science, the decision to complicate any given model or theory is often regarded as a trade-off between various epistemic values (e.g. Holman & Wilholt, 2022). For instance, sometimes simplicity matters more than predictive accuracy (or vice versa), and it is therefore unlikely that endogenisation is always epistemically more desirable. Therefore, even if one agrees with proponents of this strategy that one could, in principle, explain and predict performative changes with more encompassing and complicated models, it does not follow that such complicated models are always more adequate.

Third, endogenisation is unlikely to eliminate performative effects. For one, this is because there is no reason to assume that through endogenisation, a model suddenly becomes immune to becoming performative itself again in ways that the model does not account for. Conceivably, this might be countered with additional attempts at endogenisation, but this re-instantiates the very same problems listed here and even opens the door to an infinite regress towards ever larger models. For second, there are conceptual reasons that make an elimination-by-endogenisation unplausible, which I will get to later.

Lastly, and most importantly, even if science could eliminate its performative effects by adopting the strategy of endogenisation, this strategy does not exempt it from the political burden that has been introduced with performativity’s potential of target transformation in the first place. This argument has forcefully been made by Khosrowi (2023, 385). If the threat of performative paternalism is that it burdens scientists to illegitimately select for different outcomes, then choosing endogenisation and the specific outcome that comes with that choice does not, in fact, relieve any of that burden. When we let scientists deliberately deselect performative outcomes on the grounds of e.g. prioritising predictive accuracy, analogous questions of legitimacy arise. To endogenise or not to endogenise remains a choice and a value-laden one at that.^11^

Thus, although endogenisation appears initially attractive, given the above reasons, it is unlikely effectively address the worry of performative paternalism. It does not, at any rate, absolve scientists from having to make value-laden and potentially paternalist choices.

### Orthogonality

Likewise recognising a shortage of workable options, Khosrowi proposes another attempt to keep illegitimate value judgements by scientists at bay. He calls it the principle of *orthogonality*.

*[O]rthogonality requires that the choices modelers make in constructing and using models be robust over changes in their views on the desirability of certain social outcomes. (*Khosrowi, 2023*, 386)*

In the COVID-19 case, Khosrowi argues, orthogonality requires that scientists make their modelling decisions independently of whether they think more aggressive or milder mitigative measures would be appropriate. On my reading of Khosrowi, choosing whether to endogenise or not should thus be based purely on epistemic values, with no consideration of whether this choice will foreseeably lead to good or bad outcomes. Non-epistemic considerations of performative outcomes, I understand, are to be deferred to decision-makers that are democratically (or otherwise) justified to make such decisions. He proposes that there are institutional designs which could conceivably help achieve such a clearer division of labour between political decision-makers and modellers, facilitating transparency and so on.

However, apart from the fact that, as Khosrowi concedes too, this is more like an aspiration than a solution, I fail to see significant improvements compared to their *ex-post*-*only* appraisal proposal. Insisting on orthogonality, no matter whether scientists themselves expect performative downstream effects, I fear, suffers the very same fate of promoting forms of ex-ante ignorance. As I have hinted at before, ex-ante ignorance strikes as an instance of target obfuscation, too, and therefore also runs the risk of introducing illegitimate value judgements. For scientists to prioritise epistemic over non-epistemic values, as the principle of orthogonality attempts, is a non-epistemic value judgement in its own right, and a disagreeable one at that.^12^

### Conditionals & projections

Another response would be to question the performative status of certain models altogether and, in doing so, also question the extent to which it even makes sense to regard their primary purpose to be non-epistemic performativity. Specifically, in objection to previously mentioned examples, it might be questioned whether conditionals of the form “if you do X, then Y happens” can even be considered an instance of reflexive performativity if all they do is inform actors of the consequences of their own deliberate actions. Recall van Basshuysen et al.’s example of a doctor’s advice. Under normal circumstances, we would expect the doctor to have good epistemic warrants to predict what will happen conditional on the patient’s deliberate action. Importantly, however, note that their conditional knowledge about the underlying system is neither challenged by the patient’s action, nor is the system itself transformed in response to these actions in a manner that would make the doctor’s understanding of it obsolete. As such, given that the truth or target fit of the conditional remains unaffected by the patient’s actions, one might argue that it does not even classify as being performative at all (i.e. not as reflexively performative, only generically).^13^

Evidently, this is not how van Basshuysen et al. conceive of the relationship between performativity and conditionals, given that they rely on the doctor’s example to illustrate their key point (that performativity can be a legitimate model purpose). As such, they assume a more permissive notion of performativity, one which extends to conditionals and ordinary intervention, too. Yet, notice that permissiveness on this point drastically affects the feasibility of the manipulation strategy as a whole. If we include conditionals and conditional intervention as being regular instances of performativity, then defending performativity as a legitimate model purpose becomes almost trivial. This is because the ability to intervene successfully is one of the primary reasons why we want epistemically adequate models and theories in the first place.

On the other hand, if we were to reject ordinary interventions as instances of genuine performativity, then regarding performativity as a legitimate model purpose becomes significantly more futile. This appears to be the view by Winsberg and Harvard (2022, 512–513) when they put conditionals at the centre of their analysis and explicitly distinguish between *forecasts* and *projections* as the basis for their charge against van Basshuysen and colleagues. In short, according to them, while *forecasts* are predictions of what will actually occur, *projections* are predictions under selected conditions. At first sight, again, and as Winsberg and Harvard argue, this appears to offer elegant answers about scientists’ non-paternalist duties in the face of performativity: by offering true conditional projections instead of performative unconditional forecasts, scientists are not deciding on behalf of other people; they are doing their epistemic job, thus achieving the exact opposite of deceit and manipulation. Such projections take the following familiar form: If you exercise, you live longer (but it remains your decision); If you enact policy X, the incidence will be such and such (but it remains your decision). Consequently, Winsberg and Harvard cast the ability to project truthfully as a decidedly *epistemic* purpose, which makes it distinct and prior to whatever may then be left of the concept of a performative model purpose. If you claim performative effects could be more important than a model’s epistemic adequacy, the argument goes, and if you also think that performativity cannot simply be ordinary intervention, then what else are you proposing if not an attempt to justify telling people any bad lie as long as it gets them going the right way. From this vantage point, one can see how Winsberg and Harvard (2022, 515) arrive at their conclusion that such a practice can “never” be legitimate.

From this discussion, it might appear as if we are left with just two options. Either performativity extends to ordinary intervention and, therefore, is trivially justifiable as a model purpose; or performativity is non-ordinary as a model purpose, but then opens the floodgates to untenable lying and deceit. Indeed, on my reading, one major source of disagreement between Winsberg et al. and van Basshuysen et al. lies precisely in this definitional difference, yielding conflicting assessments of legitimacy. It certainly does not lie in the fact that one side openly intends to defend outright lying with false conditionals as a sound and just basis for decision-making.

Case closed? Unfortunately, not quite. Although the dichotomy above raises interesting questions about the overlap between conditionals and performativity, it arguably misses the mark of what the literature on performativity has traditionally been about, both in its Austinian origins surrounding speech-act theory as well as during the later appropriation of the term by sociologists of economists. Recall that these primarily circled around (a) the constitution of acts by means of utterances, (b) causal contributions to the creation of social structures or institutions by means of conceptualising them, and (c) the questionable aptitude of the concept of truth for such acts in the first place (see Section 2). While conditionals pose potentially problematic edge cases for the working definition of performativity I proposed in Section 2, any resolution of those is unlikely to significantly advance questions about the ethics of such constitutional or causal acts, for the simple reason that they refer to an entirely different category. As I see it, it is fully conceivable that *both* predictions and projections could, in principle, be involved in such constitutive or causal procedures, making any distinction between them only tangentially relevant.

Consider the following example. How would a conditional projection look like in the bank run case? An economist might project “if 10% of customers withdraw their money, this will trigger a bank run”. Let us assume this is a true, sound, and well-accepted counterfactual projection at time *t* for some future period *t* + *1*. If we thought that communicating such high-quality projections about the consequences of peoples’ actions is all we should ask from the economist, we could stop here. Yet, arguably, there is more to unpack.

First, note that whether there will be, in fact, 10% of withdrawals is not up to the decision of any one customer. Rather, it is contingent on the shared expectations of many about what others will do and whether the threshold will be reached. This contrasts sharply with the simpler doctor’s case, where the decision to act in a way that makes the antecedent of the conditional true (or false) has been entirely up to one single agent, the patient. Second, when economists spend significant airtime projecting bank runs, it is reasonable to assume this could heighten anxiety among customers about the probability of the threshold being reached. Taking those two points together, the dissemination of a projection, which was true at period *t,* could feasibly lead to changes within the target system such that now, perhaps only 5% of customers withdrawing would suffice to trigger a bank run at *t* + *1*. This would render the original projection inadequate and make it an instance of performativity proper.

While there is more to be said than I can cover here, let me close this point with two remarks. First, even if projections would turn out to be the preferred way to handle most instances of performativity, as opposed to my claims, there likely are many situations where this strategy remains unpractical. For example, Birch (2021, 90) reports that epidemiologists occasionally had to shift from conditional recommendations to unconditional advice (“Do X now!”), which he argues can be justified in extreme situations requiring immediate action. Second, a key question for the ongoing debates on the COVID-19 case is whether epidemiologists’ work more resembles the bank-run projection case proposed here or is more like the simpler doctor’s advice. An answer here probably requires nuance, and I do not intend to suggest one here.

As this debate has shown, however, and in defence of Winsberg and Harvard, the doctor’s analogy proposed by van Basshuysen et al. strikes as a particularly poor example to support a more general point about the (il)legitimacy of performativity as a model purpose. At the same time, neither are conditional projections alone likely to resolve the ethical and political problems posed by performativity of science, narrowly conceived.

### Summary

Let me take stock. I have examined four strategies that address the problems posed by performative paternalism, each with discernible flaws. The two flavours of the manipulation strategy, ex-post and ex-ante appraisal, appear either meaningless or unjustifiably paternalist. Endogenisation, in turn, cannot simply resolve the political burden placed on scientists by performativity and instead raises similar concerns of legitimacy. Likewise, orthogonality seems to repeat errors of self-deception, and a conditionals-only approach appears to miss the point about performativity more generally. Given these negative results, I will now turn towards a different approach. Instead of attempting to free scientists from the burden of having to make politically salient value decisions when facing performativity, I will ask whether there are conditions under which they can or should legitimately do so.

## The case for performative paternalism

To that end, I distinguish between two broad classes of cases. In Section 6.1, I highlight that there are numerous circumstances in which scientists (or other epistemic agents) are deliberately tasked with the role of deploying performative models, and they do so in a transparent and non-problematic manner. As such, these cases hardly involve deceit, and the worry of performative paternalism does not apply to them, rendering the strategy of manipulation not inherently problematic.

In Section 6.2, I shift to a class of cases in which manipulative roles are not being made overt and to which the worry of performative paternalism, therefore, does apply. However, I propose that acting paternalistically here can be justified, at least under some narrow conditions. To that end, I will introduce a case on modelling climate change mitigation efforts (Ortmann & Veit, 2023). Unlike the COVID-19 case, here it is assumed that harmful performative effects have already been present before any paternalist deliberation has been made, which renders a value-laden and paternalist decision on how to performatively change the world inescapable.

In Section 6.3 I attempt to generalise this argument towards a case in support of performative paternalism.

### Manipulation without act obfuscation

As remarked earlier, on the one hand, van Basshuysen et al. (2021) and Khosrowi (2023) explicitly reject ex-ante appraisal of performativity on the grounds that it would invite illegitimate value influences. On the other hand, somewhat confusingly, they frequently draw onto examples that appear to involve decidedly forward-looking attitudes towards performative effects while being legitimate. Recall the doctor example contested earlier. A perhaps less controversial example of genuine forward-looking performativity, which both van Basshuysen (2023) and Khosrowi (2023) invoke in later publications, is that of applied market design in economics, such as auction design. When designing auctions, economists use models to structure bidding processes, aiming to achieve certain outcomes such as maximising revenue, ensuring fairness, or enhancing market efficiency. By informing sellers and auctioneers which set of rules should be enacted, those models are not simply forecasting but actively involved in the process of creating conditions to achieve the desired effects (Callon & Roth, 2021). Oftentimes, the very same models are also used by bidders to guide their own decision making and to predict the strategic behaviour of the other bidders. The widespread enactment of auction theory is arguably seen by many in the literature as a genuine instance of forward-looking performativity.

Clearly, if legitimate and performative, both examples go against Winsberg and Harvard’s strong claim that performativity is *never* a legitimate model purpose. They also, however, go against van Basshuysen et al.’s own claims that only ex-post appraisal of performativity is permissible. This begs the question, then, what is the difference between legitimate and illegitimate attempts to elicit desirable future changes in one’s modelling target?

Cutting to the chase, one major reason seems to be that, in each of these cases, no obvious deceit is happening and, as a result, no significant infringement on autonomy. To the degree that the doctor or the market designers manipulate their targets, they do so overtly.^14^ More specifically, none of the previously identified harms associated with performative paternalism apply. To start with, there is no *act appropriation* present, nor any *act obfuscation*. It is a doctor’s professed duty to “change” things about their patients, including making decisions on behalf of their well-being, and they are doing so in a consensual manner. The same goes for economists who are mandated by a government to come up with a specific market or auction design. Second, given that there is a clear mandate in place, there is also no *target obfuscation* going on: In the doctor’s case, the patient is arguably now in a better-informed position to deliberate on their own future action. In the auction’s case, it is an obvious fact that auctions are human creations that could be designed differently, too, and there is no false sense of inevitability. Third, there is little worry about the desirability of the foreseeable *target outcome*: the patient is better informed about potential future paths, and if they indeed died early, this would not have been due to obtrusive value judgments by the doctor. Similar things hold for the economist “engineers”, where the desirability of the outcome is vouched for by the government.

As such, the above cases can hardly be considered paternalistic. Between doctor and patient, usually, there is a direct relationship of consent. Between economists and market participants, the relationship is more complicated and not necessarily consensual, but at least their performative actions are democratically legitimised given that their designed setup is usually enforced and endorsed by the policymaker. Hence, to the degree that any autonomy is given up in these cases, it is being done in a justifiable manner.

Naturally, however, not all scientists who deploy potentially performative models are in such well-defined and well-justified roles. In fact, the opposite is arguably true: performativity is usually taken to create problems for scientific activities precisely because it does not sit well with commonly assumed societal roles of researchers. Yet, in the following, I will argue that the absence of such an explicit role does not, in principle, inhibit the justification of pursuing the manipulation strategy.

### Manipulation with act obfuscation

Consider the following case by Matthew Kopec (2017), along with a response that Walter Veit and I have offered (Ortmann & Veit, 2023). According to Kopec, the lack of internationally coordinated climate change mitigation efforts might partly stem from a self-fulfilling prophecy. This is because climate change mitigation has become a paradigm case for the model of the *Tragedy of the Commons* (ToC), a game-theoretic model that casts a particularly dismal picture of our ability to alleviate a climate crisis. It depicts climate change mitigation as a situation in which everybody involved has an individual incentive to keep emitting more, irrespective of what the others do, with the effect that the common resource of a greenhouse-gas-free atmosphere will be depleted and the mutual benefits of jointly limiting emissions are not reaped. The rough idea is this: If everyone else started mitigating emissions, I would benefit from continuing to emit because I would not need to invest in a costly energy transition. Likewise, if the others do not mitigate either, then the climate crisis is happening anyway, and I can keep emitting more, too.^15^ Importantly, given the nature of the collective action problem of climate change (it is geographically and temporally dispersed), all well-trodden ways of dealing with ToC-like situations are hard or impossible to implement (e.g. a global carbon prize).

According to Kopec (2017), the ToC model exhibits certain features that plausibly render it to be a self-fulfilling prophecy (see also MacKenzie, 2006, 43—46). For example, the ToC is relatively simple while, at least at first sight, also providing significant explanatory depth. Perhaps most importantly, it also has been credited with a high degree of scientific approval – in a summary for decision-makers, for instance, the IPCC (2014, 211) ascribed “high confidence” to the claim that climate change is a ToC. The concern raised by Kopec, then, is that if decision-makers conclude based on the ToC that their dominant strategy is to keep emitting, no matter what, then climate change mitigation, in fact, does become a ToC.

Surely, if nothing else, a self-fulfilling ToC is even more tragic than the original already is. In response, Walter Veit and I have previously proposed what is essentially an instance of what was here called the manipulation strategy (Ortmann & Veit, 2023); in short, under the condition that the ToC is indeed self-fulfilling, scholars would have both ethical and epistemic reasons to switch to more optimistic ways of modelling climate change with the specific purpose of fostering international cooperation levels. The argument goes as follows. By and large, the ToC model depicts two options by which an agent such as a nation-state is able to take action: they can decide to emit either “more” or “less” greenhouse gases. Alternative models emphasise a different or larger range of options for modelled agents. For example, joint climate change mitigation has been modelled as a series of interactions in which the necessary level of cooperation can evolve over time. Other perspectives have highlighted the multiplicity of co-benefits that come with sustainable divestment, such as significantly lower energy costs, reduced dependence on oil-exporters, technological supremacy, and so on, which also could render it individually rational to step up mitigation efforts. No matter which option sounds most plausible at face value, the important bit is any choice between them is *underdetermined* by the available evidence. As such, any choice that is being made for or against any such model is inherently value-laden.^16^ Loosely summarising, there are two main sets of values at play here for model selection.

The first set concerns the epistemic adequacy of the ToC model. Under the condition that the ToC is, in fact, a self-fulfilling prophecy, then it is already implied that the ToC does not capture all the relevant causal vectors that lead to the observed lack of cooperation. As such, the ToC model already has apparent flaws as a descriptive model, which one might consider “reason enough to justify […] emphasising […] alternative or more refined explanations “ (Ortmann & Veit, 2023, 20). This is consistent with the more generalised way in which performativity has been pictured here previously. Recall that reflexive performativity, as defined in Section 2, occurs if and only if models interact with their targets such that the interaction bears on model-target-fit. Thus, whenever it is established that a phenomenon is subject to performativity, it has already been granted that modelling practices are among the relevant causes of observed changes. As a side note, recall that a similar consideration initially motivated the previously discussed endogenisation strategy. As Laimann (2020, 1056) had put it, performativity “rubs our nose in the fact” that the models we are using “are based on an inadequate understanding of the phenomena in question.” Thus, if performativity renders the model in question sufficiently inadequate from an epistemic point of view, this provides a first rationale for deselection.

The second set concerns the consequences of scientists’ assertions (such as the IPCC prominently asserting the ToC). Accordingly, the existential risk associated with the climate crisis justifies heightened scrutiny not only of the overall adequacy of the ToC as a model, but also of the scientists’ role in potentially contributing to it (Ortmann & Veit, 2023, 66–67). If it is plausible that scholars of international relations actively contribute to fueling a global emergency due to their decisions of how they frame it, this deserves serious consideration. And if they do, there is a case to be made that they ought to cease doing so and deselect the ToC as a model.^17^

At any rate, this argument fits the criteria that originally had given rise to the worry of performative paternalism: It is an instance of the strategy of manipulation featuring deliberate ex-ante appraisal of performativity, where the performative effects of emphasising alternative models (be that endogenising models or others) are deliberately taken into account to inform a value-laden model choice. The justification for this deselection, however, is distinct from previous examples; as it happens, scholars of international relations are not usually regarded as occupying overtly executive positions similar to that of medical doctors or economists-as-engineers, and neither does this argument assume such a role to be necessary for justifying deselection. Instead, I contend, if the ToC is indeed a self-fulfilling prophecy, deselection can be justified despite being paternalist. If correct, this case may function as a counterexample against the position that performative paternalism could *never* be justified.

Drawing on the previous discussions, I aim to work out some details of this argument and generalise it. The main justification for this case, I take it, rests on an as of yet implicit point that making a performative choice here is inescapable. Let me attempt to spell this out in more detail.

### Justifying performative paternalism

Notice that in the ToC case, it is assumed that harmful performative effects are already present; Kopec worried that the ToC is currently in the process of enacting a self-fulfilling prophecy. This had not been assumed for the COVID-19 examples, where the discussion revolved around model appraisal as either ex-ante or ex-post of model deployment and was not conceived as a concurrent phenomenon. While this might seem like a subtle point, as I will show now, it has important implications regarding a scientist’s generally available options. In short, if performative effects are already present, making *some* choice in a paternalistic way is, I argue, inescapable.

Premise 1: *If performative effects are already present, the exhaustive set of choice options a scientist is left with consists of the following three options*. Option 1 is to continue using the performative model in question. Option 2 is to suspend a judgment and refrain from communicating any model or prediction. Option 3 is to deselect the current model and replace it with another one – either an endogenising model or a non-endogenising one. While a scientist might switch between those options over time, or combine them for different target audiences, they can only ever choose among these.

Note that the previously discussed cases of *ex-ante ignorance* can be instances of any option 1 to 3. If *orthogonality* or *ex-post-only* imply that model selection is irresponsive to the insight that the model in question is performative, then this can entail anything between continuing to use the model, suspending any judgment, or choosing a different model.

It might be objected that a fourth option exists: for each of the above three options, a scientist could attempt to disclose their actions, e.g. to seek public approval. This would work towards transforming the scenario towards a non-paternalist one, as highlighted in Section 6.1. First, however, as remarked earlier, we should expect that this is not an available option in every case. The executive summary of the IPCC report, for example, is called a *summary* for a reason. Putting an elaborate endogenising story here that discloses the likely performative effects of the report itself would surely seem misplaced. I thus proceed by assuming a case for which the above three options are exhaustive. Second, as I see it, disclosure arguably collapses towards option three if that involves an alternative endogenising explanation.

Premise 2: *If performative effects are already present, any one of those options can be considered to be performative*, meaning they causally affect the target in a way that bears on model-target-fit. Regarding option 1, continuing to use a performative model simply continues its effects and thus is performative by construction. Regarding option 2, while a suspended judgment is not strictly speaking performative as defined earlier (recall that the definition of reflexive performativity involved models, theories, or predictions, not their absence), a suspended judgment can also incur downstream effects which can bear on model-target-fit of the initial model.^18^ For example, epidemiologists could have decided not to publish any predictions whatsoever, taking away resources for public decision making, which, conceivably, would have altered the pandemic, too. Regarding option 3, deselecting a performative model and replacing it with a new one would either introduce another performative model, or one would choose a model for which no further performative effects are expected. Perhaps this would “push” away the target from how it is currently constituted towards how it would have been had the initial performative model not been in place to begin with. However, as such, this remains a decision bearing on model-target-fit and, therefore, is performative, too.

Premise 3: *If performative effects are already present, any of those options is paternalist*. Recall the definition of paternalism from earlier. The two main conditions were that (a) the decision is made non-consensually and (b) on behalf of the well-being of others, which both appear to be fulfilled for each of the three options. Usually, model decisions and science communication are sufficiently intricate such that it is simply impossible to gather the consent of everybody potentially affected by a performative model's effects. Pandemic models are a case in point. Furthermore, apart from the fact that model choice between alternative candidates is value-laden because it is underdetermined by evidence (as Ortmann and Veit (2023) argued), it is also value-laden in the sense that this remains to be a choice *for* a social outcome (as Khosrowi (2023) argued). Given that model choice carries these value judgements at least implicitly, including attempts of disclosure, this also remains a decision on behalf of the well-being of others and, therefore, paternalist. If, for example, proponents of the ToC decide that epistemic values (such as simplicity of the model) matter more than the prospect of contributing to a deteriorating cooperative environment, this is a non-democratic paternalist choice of what they think matters more.

Premise 4: *If performative effects are already present, at least one option is justified at any given moment*. This is mainly because under the condition that the set of three options is indeed exhaustive, scientists need to choose at least one of those options. Which one, however, is likely to be contingent. Recall that the main departure point of this paper has been that deliberate deployment of performative models causes particular harms. The same, however, can be said about continued adherence to a performative model once it has been established that performative effects are happening. Take the ToC case again. Regarding *target outcome*, fueling the climate crisis via performative models is clearly undesirable.^19^ Regarding *target obfuscation*, the tragedy of the commons model casts the climate crisis as a rational necessity (Kopec, 2017, 217–218), which is a shaky claim at best once we consider alternative perspectives that also predict higher cooperation levels, and especially if we think that it is the model itself that takes part in making itself true. Regarding *act obfuscation*, scientists pretend to provide a simple and accurate model, while in reality, they reinvigorate a performative model. Regarding *act appropriation*, scientists should not be in a position to fuel the climate crisis. Whichever of the three options is justified can thus be expected to be context-dependent, but at least one must be chosen.

Conclusion: *If performative effects are already present, performative paternalism is justified*.

## Conclusion

In this paper, I have agreed with van Basshuysen et al. (2021) that the strategy of manipulation can, in principle, be justified, but I have argued that any such justification needs to cut at a different joint. Specifically, I contended that wherever performative paternalism strikes as problematic, it is not because of its forward-looking nature but, among others, because of deceitful obfuscation of the scientist’s true act. The wrongfulness of deceit, however, cuts both ways, which makes obfuscation or ignorance towards already existing harmful performative effects wrongful, too. I have thus concluded that if performative effects are already present, performative paternalism is inescapable and justifiable.

More broadly, this has been an attempt to consolidate existing coping strategies, namely the strategies of endogenisation, manipulation, orthogonality and projection. If performativity is indeed inescapable in some situations, responsible management of performativity cannot be about *whether* any coping strategy introduces value influences by scientists and how these can be warded off – this is what, in my eyes unsuccessfully, both the orthogonality and ex-post-only approaches have attempted. Instead, it is about asking *which* value judgments by scientists are legitimate and how scientists can handle performativity neither with deceit nor with ignorance. I see this argument to be in broad alignment with a more general trend in the values in science literature to pivot away from the question of *whether* value judgements play important roles towards asking *which* value judgements are the legitimate (Holman & Wilholt, 2022).

If my argument is correct, a main task for responsible handling of performativity in science consists of determining whether, in a given situation, performative effects are, in fact, already present or not, or whether there are other reasons that might render performativity inescapable. I do not take any stance towards whether this question has been answered for epidemiological cases, but I do think that Kopec (2017) has made this case convincingly regarding climate change and the ToC. If this turns out to be the case, another task could consist of investigating whether a particular scenario could not be transformed towards one of the unproblematic cases of non-deceitful performativity, such as auction design.

Moreover, the proposed argument aligns with a number of approaches in the recent performativity literature, broadly construed. Koskinen (2022), for example, argued that performative effects could fruitfully and legitimately be used by self-proclaimed activist researchers as a tool to replace existing harmful social structures in an act of “mental decolonisation”. I agree and take this to be justifiable by two reasons discussed here: there is little act obfuscation (activist researchers are open about their intents), and harmful colonial performative effects are presumed to have already been in place. Another argument I want to highlight is by Godman and Marchionni (2022), who argued that scientists have a duty to prevent foreseeable harms caused by their performative models. While they have focused on the ways in which institutional design can help to align such harm prevention with the actual interests of those affected, I have here focused on the potentially paternalist character of such decisions, which does not inherently stand in the way of their legitimacy. Lastly, Hilligardt (2023, 134) argued for a “pluralist system of scientific mandates”, which need not be restricted to pursuing only democratically agreed-upon aims. Given that the nature of model choice and performativity in science may render democratic model choice unfeasible, I agree and find that the case for performative paternalism supports Hilligardt’s case.

The sound of acting paternalistically, I admit, does not initially have the greatest appeal and is nothing scientists should strive for, generally speaking. Given the nature of performativity, however, we need to find strategies that do not repeat the harms they seek to solve, i.e. scientists illegitimately imposing their value judgements on society. Accepting that this entails forms of paternalism, as I have tried to argue here, does not open the door to deceit but is, in fact, necessary to set up a transparent, non-ignorant deliberation process about both the performative roles scientists ought to occupy in society and about the ends that should be achieved by means of relying on performative models.

## Competing interest

The author has no financial or non-financial interests that are directly or indirectly related to the work submitted for publication.

## Footnotes

1 By scientific conceptualisations, I mean theories, models, explanations, predictions, classifications, etc. Given their shared use as epistemic tools that influence expectations of how a system might be behave in the future, the phenomenon I am interested in applies to all of them.

2 Related terms are *reflexivity* (Bourdieu 2004; Northcott 2022; Soros 1987), *looping effect* (Hacking 1986), *Pygmalion effect* or *Rosenthal effect* (Rosenthal 1976), *reaction to public prediction* (Grunberg and Modigliani 1954), *autogenetic processes* (Kukla 1994), *word-to-world relationships* (Searle 1983) and more.

3 This classification is based on MacKenzie (2006), but only roughly. First, his examples of “generic performativity” all come from economics and it is not clear to me whether he would agree with the nuclear fission example. Second, instead of “reflexive”, he suggests the term “effective performativity”, which falsely suggests that generic performativity could not also have normatively or epistemologically salient downstream effects.

4 This is not to say that generic performativity cannot come with ethical complications, too (Franco 2017, 2019).

5 Note that the terms *performativity* and *performance* as used here have nothing to do with each other. This is not self-explanatory, given other uses of the term (e.g. Butler 1990).

6 Mario Draghi (2012), then president of the European Central Bank (ECB), became famous for supposedly saving the Euro single-handedly with a speech in which he assured the ECB would “do whatever it takes” to save the currency.

7 Loosely inspired by Dworkin (2020).

8 One could also think of instances where the manipulation strategy is deployed with the purpose of decreasing others’ well-being. I do not discuss such cases and assume good intentions.

9 In this sense, the term *endogenisation* is misleading. Including performative effects as an *exogenous* variable, meaning that they are not explained by the model itself but taken as a given, would still count as an instance of pursuing what I here treat as the endogenisation strategy in a broader sense. Because this terminological disagreement is insignificant towards my main argument, I will bracket if off here.

10 Epidemiology, however, is by far not the only discipline that has attempted endogenisation as a way to counter performativity. For instance, similar arguments, although with different terminology, have been made surrounding the debates of Ian Hacking’s looping effects of human kinds. See Jessica Laimann (2020) for an excellent example of proposing what I call endogenisation here.

11 See also Law and Urry (2004, 404) for a similar argument: “to the extent social science conceals its performativity from itself it is pretending to an innocence that it cannot have.“ See also Kukla (1994) for a related point.

12 See Brown (2013) for a related argument (made outside the context of performativity) that lexical priority of epistemic over non-epistemic values encourages illegitimate value influences.

13 I thank Ahmad Elabbar for pointing this out to me.

14 That is, if all goes well. As one anonymous reviewer rightfully pointed out, there is no guarantee that economists did not play a non-overt role in determining targeted outcomes.

15 This reproduction of the Tragedy of the Commons idea is more akin to the more simple Prisoner’s Dilemma, but for this argument their differences are not too relevant.

16 See our previous paper for the full argument (Ortmann and Veit 2023). See also Stanford (2023) for a more detailed treatment of underdetermination in general.

17 One might object that the tragedy of the commons is more likely to have the opposite effect, or that it is designed to portray cooperation as desirable. We consider this option in Ortmann and Veit (2023) and follow an argument by Northcott and Alexandrova (2015): If the model is descriptively false and merely used to show that we ought to collaborate, then people would behave differently anyway and the normative advise would miss its target.

18 See e.g. Kukla (1994) who considers this option.

19 Ignoring individuals here that benefit from continued carbon fuel dependency.
